# PRImus‐AD study: Design of the phase 2 study treating patients with MCI or mild dementia due to Alzheimer’s disease (AD) with the orally available PRI‐002

**DOI:** 10.1002/alz70859_103102

**Published:** 2025-12-25

**Authors:** Dagmar Jürgens, Gerhard Tischler, Alexander Brener, Tina Bartsch, Gunther Kauselmann, Knut Adermann, Kathrin Zeiger, Katja Lindner, Julie‐Anne Gabelich, Dieter Willbold, Oliver Peters

**Affiliations:** ^1^ Priavoid, Düsseldorf, NRW Germany; ^2^ PRInnovation, Düsseldorf, NRW Germany; ^3^ Forschungszentrum Julich, Julich, Priavoid GmbH Germany; ^4^ Department of Psychiatry and Neurosciences, Charité Universitätsmedizin Berlin, Berlin Germany

## Abstract

**Background:**

The drug candidate PRI‐002 is an all‐D‐enantiomeric peptide and was developed to disassemble toxic Aβ oligomers into harmless Aβ monomers. It is anticipated that PRI‐002 reduces neurotoxicity and restores synaptic plasticity in early stages of AD. In studies in healthy volunteers and in patients with mild cognitive impairment (MCI) and mild dementia due to AD (EudraCT 2020‐003416‐27) the orally administered PRI‐002 has demonstrated to be safe and well tolerable.

**Method:**

The PRImus‐AD study (EU clinical trial number 2022‐503148‐41) is a randomized, double‐blind, placebo‐controlled study to assess safety and efficacy of PRI‐002 in patients with MCI or mild dementia due to Alzheimer’s Disease (AD). The trial is conducted in six European counties at 40 study sites. The trial is registered at euclinicaltrials.eu/search‐for‐clinical‐trials/?lang=en&EUCT=2022‐503148‐41‐00 and clinicaltrials.gov/study/NCT06182085. CSF or Amyloid‐PET is used to confirm AD pathology. Stratification factors are disease state (MCI or mild dementia) and APOE e4 status. Eligible patients were randomly assigned (1:1:1) to receive 300 or 600 mg PRI‐002 per day or placebo following an adaptive design for 48 to 96 weeks of treatment duration. A follow‐up assessment is planned 12 weeks after the end of treatment. Besides to the endpoint of primary safety objectives the efficacy will be assessed by the change from baseline to week 48 in global outcome as measured by CDR‐SB.

**Result:**

540 patients in total were screened. Screening was accomplished three months ahead of the original schedule. A Screening Failure Rate of 42 % resulted in approx. 300 eligible patients (at the date of abstract submission) randomly assigned to 300 or 600 mg PRI‐002 per day or placebo. A blinded sample size recalculation confirmed the initial calculation of subjects to be enrolled. Although PRI‐002 is not expected to induce ARIAs, ARIA monitoring is closely performed for the first 90 patients until week 24.

**Conclusion:**

The PRIMUS‐AD phase 2 study will finally enroll about 300 patients with MCI and mild dementia due to AD to assess safety and therapeutic benefit of PRI‐002. Results are expected mid‐2026.

